# The potential role of Mucoromycotina ‘fine root endophytes’ in plant nitrogen nutrition

**DOI:** 10.1111/ppl.13715

**Published:** 2022-05-30

**Authors:** Nathan Howard, Silvia Pressel, Ryan S. Kaye, Tim J. Daniell, Katie J. Field

**Affiliations:** ^1^ Plants, Photosynthesis and Soil, School of Biosciences University of Sheffield Sheffield UK; ^2^ Department of Life Sciences Natural History Museum London UK

## Abstract

Mycorrhizal associations between fungi and plant roots have globally significant impacts on nutrient cycling. Mucoromycotina ‘fine root endophytes’ (MFRE) are a distinct and recently characterised group of mycorrhiza‐forming fungi that associate with the roots of a range of host plant species. Given their previous misidentification and assignment as arbuscular mycorrhizal fungi (AMF) of the Glomeromycotina, it is now important to untangle the specific form and function of MFRE symbioses. In particular, relatively little is known about the nature of MFRE colonisation and its role in N uptake and transfer to host plants. Even less is known about the mechanisms by which MFRE access and assimilate N, and how this N is processed and subsequently exchanged with host plants for photosynthates. Here, we summarise and contrast the structures formed by MFRE and arbuscular mycorrhizal fungi in host plants as well as compare the N source preference of each mycorrhizal fungal group with what is currently known for MFRE N uptake. We compare the mechanisms of N assimilation and transfer to host plants utilised by the main groups of mycorrhizal fungi and hypothesise potential mechanisms for MFRE N assimilation and transfer, outlining directions for future research.

## 1 INTRODUCTION

Nitrogen (N) is a key macronutrient in plant nutrition (Evans, 1989) and, as a result of global N limitation (Vitousek & Howarth, 1991), is a key driving force in plant competition and evolution (Kang et al., 2015; Pankoke et al., 2015). N occurs within soils in both inorganic mineral (e.g. ammonium and nitrate salts; Matsumoto et al., 2000) and organic forms (derived from plant, animal and microbial decay; Greenfield, 2001); organic N can be as high as 90% of total soil N in some habitats (e.g. moorland soil in the vicinity of *Calluna vulgaris*; Abuarghub & Read, 1988). Major classes of organic N‐containing compounds found within soils include free amino acids, polypeptides and proteins, purines, pyrimidines and vitamins. There is evidence that organic N is important for many plant N budgets across a variety of ecosystems as plant‐available inorganic N pools are often limiting (Talbot & Treseder, 2010).

>500 My of plant evolution has driven a huge array of plant adaptations and strategies to enhance N access and assimilation in N‐limited environments, including in extreme cases carnivory to directly access different N pools (Bott et al., 2008; Roberts & Oosting, 1958). In less extreme and more widespread cases, symbiotic associations with soil microbes provide indirect access to otherwise unavailable soil N pools (Phillips et al., 2011; Smith & Read, 2008). A diverse array of microorganisms live within the rhizosphere and surrounding soil, and play an important role in plant N nutrition through cycling and degradation of mineral and organic N (Phillips et al., 2011; Truu et al., 2020) and some by forming symbioses with plants. For example, rhizobia colonise the roots of legumes, inducing nodule formation, which facilitates atmospheric N_2_ fixation by the bacteria and transfer of N_2_ to the host plant in return for plant‐fixed carbon (C) (Andrews & Andrews, 2017). A different nutritional symbiosis is formed between the vast majority of plants and certain groups of soil fungi; these partnerships are known as mycorrhizas (Brundrett, 2009; Brundrett & Tedersoo, 2018). Mycorrhizal fungi have until recently been classified into four main groups based on colonisation structures, morphology, and host range (Brundrett, 2009; Brundrett & Tedersoo, 2018; Table 1), all of which show evidence of nitrogen transfer from the fungi to the host plants (Field et al., 2019; Fochi et al., 2017; Makarov, 2019; Stuart & Plett, 2020). The most commonly occurring groups of mycorrhizal fungi are spread across three fungal phyla: Mucoromycota, Ascomycota, and Basidiomycota (Spatafora et al., 2016; Stuart & Plett, 2020). Within Mucoromycota, arbuscular mycorrhizal fungi—the most researched group of mycorrhiza‐forming fungi—are found within the subphylum Glomeromycotina (syn. Glomeromycota) (Schüßler & Walker, 2011). Glomeromycotina arbuscular mycorrhizal fungi (AMF) are estimated to form associations with ~72% of vascular plants (Brundrett & Tedersoo, 2018). Recent molecular, cytological and physiological evidence suggests another group of widely occurring (Orchard, Standish, et al., 2017), mycorrhiza‐forming (Hoysted et al., 2019) fungi should now be considered alongside these; ‘fine root endophytes’ of the subphylum Mucoromycotina, within the Mucoromycota (Spatafora et al., 2016; Orchard, Hilton, et al., 2017; Walker et al., 2018; Table 1).

**TABLE 1:** Summary of key points to compare and contrast the different mycorrhizal types using the extensive literature on non‐MFRE mycorrhizal types, and comparatively sparse literature on MFRE

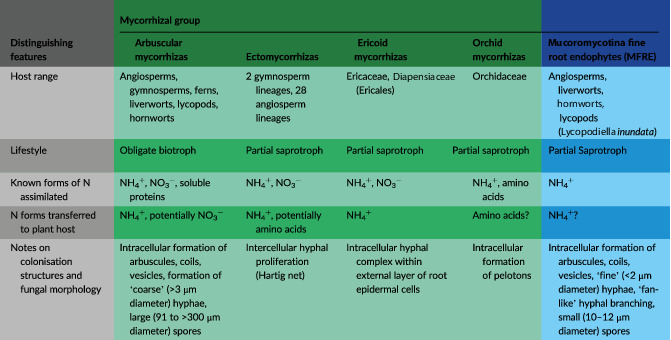

*Note*: Columns in green indicate the four traditional groupings of mycorrhizal fungi. The blue column shows Mucoromycotina ‘fine root endophyte’ (MFRE) as a fifth mycorrhizal group to be considered along with the four existing groups (Brundrett, 2009; Brundrett & Tedersoo, 2018; Chalot & Brun, 1998; Fochi et al., 2017; Hoysted et al., 2019; Rimington et al., 2020; Smith & Read, 2010; Sinanaj et al., 2021).

Despite their distinctive fine hyphae and colonisation morphology, the production of arbuscule‐like structures in planta by Mucoromycotina ‘fine root endophytes’ (MFRE) led to their misclassification as AMF within the subphylum Glomeromycotina (Orchard, Standish, et al., 2017). Following application of novel molecular detection and identification methods across a range of host plants (Bidartondo et al., 2011; Orchard, Hilton, et al., 2017) and subsequent reclassification (Spatafora et al., 2016), MFRE were recognised as a distinct symbiotic fungal type (differences detailed in Table 1). Experimental evidence suggests that, like AMF, MFRE play an important role in facilitating plant nutrient uptake in return for plant‐fixed carbon from their host (Hoysted et al., 2019). Unlike obligately biotrophic AMF (Lin et al., 2014; Smith & Smith, 2011), MFRE are thought to be facultatively saprotrophic in nature (Field et al., 2015; Field et al., 2016; Lin et al., 2014), as evidenced by isolation and in vitro culture experiments (Field et al., 2015) whereby MFRE proliferate on synthetic media without a host plant. These apparently saprotrophic qualities, together with their frequent co‐colonisation of host plants with AMF, open the possibility that MFRE may play an important role in plant nutrition that is distinct from AMF.

Recent experimental evidence in both non‐vascular liverworts (Field et al., 2019), and in a vascular plant (Hoysted et al., 2019, 2021), suggests that there is a degree of complementarity between MFRE and AMF function, with MFRE playing a more prominent role in facilitating plant N nutrition alongside AMF‐acquired P (Field et al., 2019). This hitherto unappreciated complementarity may have caused a potential source of confusion so far, whereby fungal‐mediated transfer of N to host plants has been misattributed as being wholly due to AMF. Thanks to the use of specific MFRE primers (Bidartondo et al., 2011; Desirò et al., 2017), consideration of a more cosmopolitan fungal endophyte community is now possible.

Owing to the dearth of research into the functions and mechanisms of MFRE compared to AMF, in this review we draw on existing bodies of research into MFRE, AMF, and other groups of mycorrhizal fungi in order to compare and contrast the function and potential role of MFRE in plant N nutrition with those of other symbiotic fungi. We review the current literature regarding molecular mechanisms of nutrient exchange and how soil environment may affect mycorrhizal nutrient exchange. Against this background, we pose important outstanding research questions (Box 1) and put forward hypotheses for the nutritional functions of MFRE in relation to host plants.

BOX 1 **Key outstanding research questions**

- What are the key intracellular symbiotic structures formed by MFRE for nutrient exchange, what determines their development and
- phenology?
- What is the range of nitrogen sources MFRE can access and assimilate, how does this contribute to host plant nitrogen nutrition and how does it compare to other symbiotic fungi?
- By what mechanisms do MFRE take up and assimilate nitrogen from soils?
  - Does the uptake and assimilation mechanism differ when MFRE are presented with organic versus inorganic nitrogen sources?
  - How and in what form/forms is nitrogen translocated along MFRE hyphae and into host plants?
- Does the form of nitrogen assimilated by MFRE affect fungal acquisition of plant‐assimilated carbon from host plants?
- How do the absolute concentrations of soil nitrogen and phosphorus, as well as N:P ratio affect MFRE‐mediated plant nutrient acquisition?
- What is the role of soil microbes in MFRE‐mediated nitrogen uptake by host plants?

## 2 FUNGAL ORGANS OF NUTRIENT EXCHANGE

**FIGURE 1:**
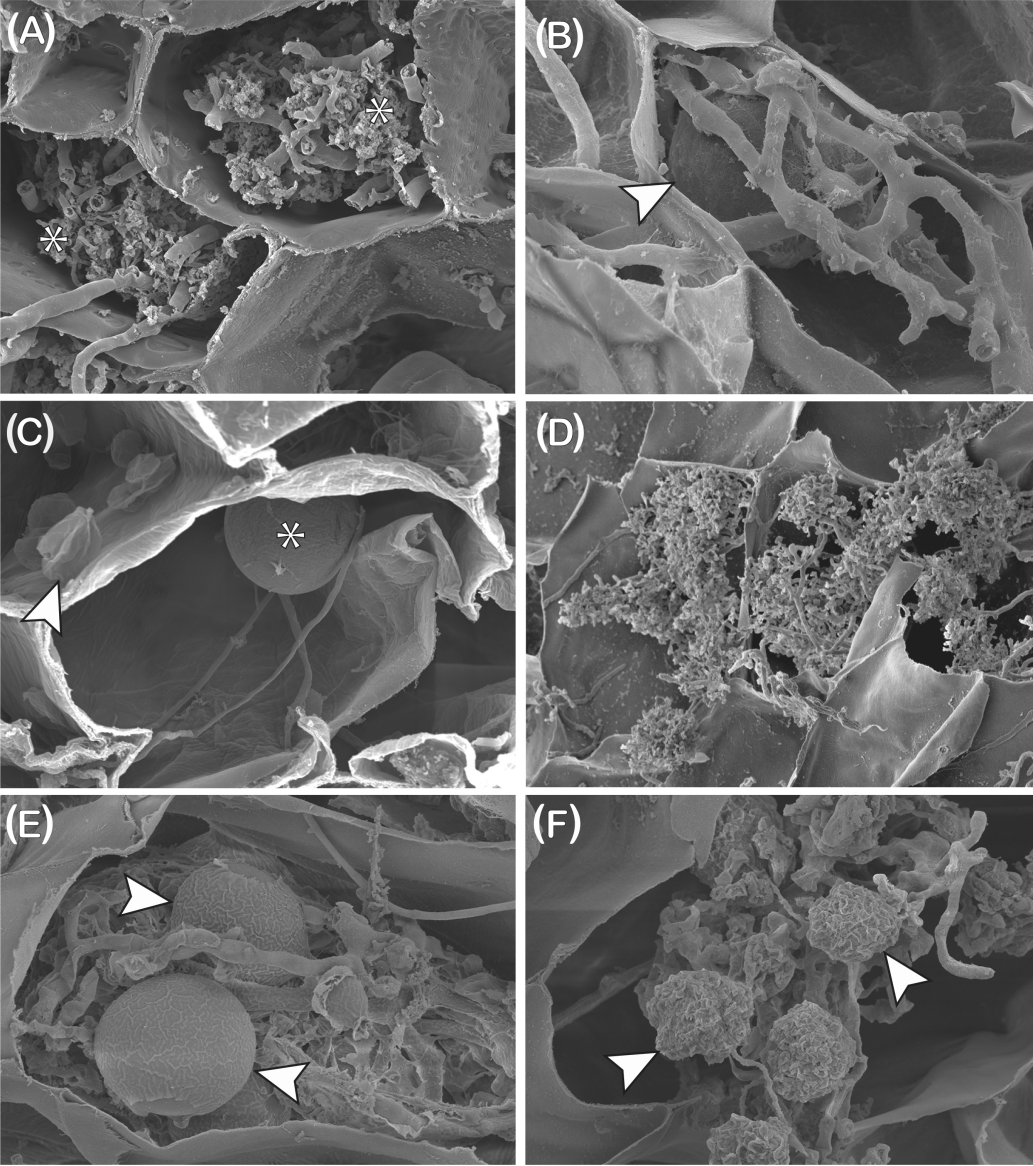
Scanning electron micrographs of structures produced by AMF (A,B) and MFRE (C–F) during colonisation of host plant tissues. (A,B) Typical arbuscular mycorrhizal fungi (AMF) intracellular ‘coarse’ arbuscules (A, arrowed) and large vesicle (B, arrowed) with coarse hyphae, shown here in the thallus of the liverwort *Neohodgsonia mirabilis*. (C) Mucoromycotina ‘fine root endophyte’ (MFRE) intracellular intercalary and terminal small vesicles/swellings, shown here at different stages of development (young*; collapsed, arrowed) with fine hyphae in a root of the vascular plant *Lycopodiella inundata*. (D) Intracellular fine arbuscules (arrowed) in the liverwort *N. mirabilis*. (E,F) Intracellular tightly wound hyphal coils with young (E, arrowed) and collapsed (F, arrowed) ‘lumps’ in the thallus of the Haplomitriopsida liverwort *Treubia lacunosa*. Scale bars: (A, B, D) 20 μm; (C, E, F) 10 μm

The colonisation morphology of different mycorrhizal fungal groups is highly characteristic and is widely used as the basis by which they are distinguished from one another without molecular characterisation and vice versa (Brundrett, 2009; Brundrett & Tedersoo, 2018; Table 1). It is important to note that the extent and types of the fungal structures that form within plant roots during mycorrhizal symbioses are highly dependent on the species of both plant and fungus (Dickson, 2004). AMF form characteristic structures within the root cells of their host plants. These include arbuscules that are terminally differentiated, highly branched structures that provide a large surface area for nutrient transfer between symbiotic partners, vesicles for lipid storage, and spores for lipid storage and propagation (Luginbuehl & Oldroyd, 2017; Figure 1). Ectomycorrhizal fungi (EcM) colonise the lateral roots of their host plants by forming an interlacing mycelial structure, known as the Hartig net, which penetrates between and surrounds the epidermal cells (Stuart & Plett, 2020) and, like the arbuscule, provides a large surface area for nutrient exchange. However, unlike AMF arbuscules, plant cell walls are not penetrated during the formation of the Hartig net. This is compensated for by the development of lateral root clusters and tubercles that increase the interface surface area (Smith & Read, 2008). Roots of ericoid mycorrhizal fungi (ErM)‐colonised plants are highly specialised; they lack root hairs and have very narrow diameters (100–<50 μm in distal regions). The roots have a single layer of epidermal cells that are colonised by ErM; typically, the cell wall is penetrated once and a hyphal complex occupies the whole cell (Smith & Read, 2008). All orchid species rely on orchid mycorrhizal fungi (OrM) at some stage during their life cycle. Owing to their small seed size and lack of sufficient nutrition, OrM are required for growth of orchids beyond the protocorm stage of development into adulthood (Smith & Read, 2008). In both the protocorm and adult orchid roots, the fungus forms pelotons through the growth and anastomosis of intracellular hyphae after cell wall penetration, increasing the interfacial area between fungus and plant (Smith & Read, 2008). Individual pelotons have a duration of less than 2 weeks (Mollison, 1943) at the end of which they collapse and are digested, which is traditionally proposed as the main mechanism for nutrient transfer to the host plant (Fochi et al., 2017; Smith & Read, 2008). However, although fungal lysis may contribute to some plant nutrient gain, there is now convincing evidence of transfer across intact membranes as in other mycorrhizal symbioses (Fochi et al., 2017; Kuga et al., 2014).

**FIGURE 2:**
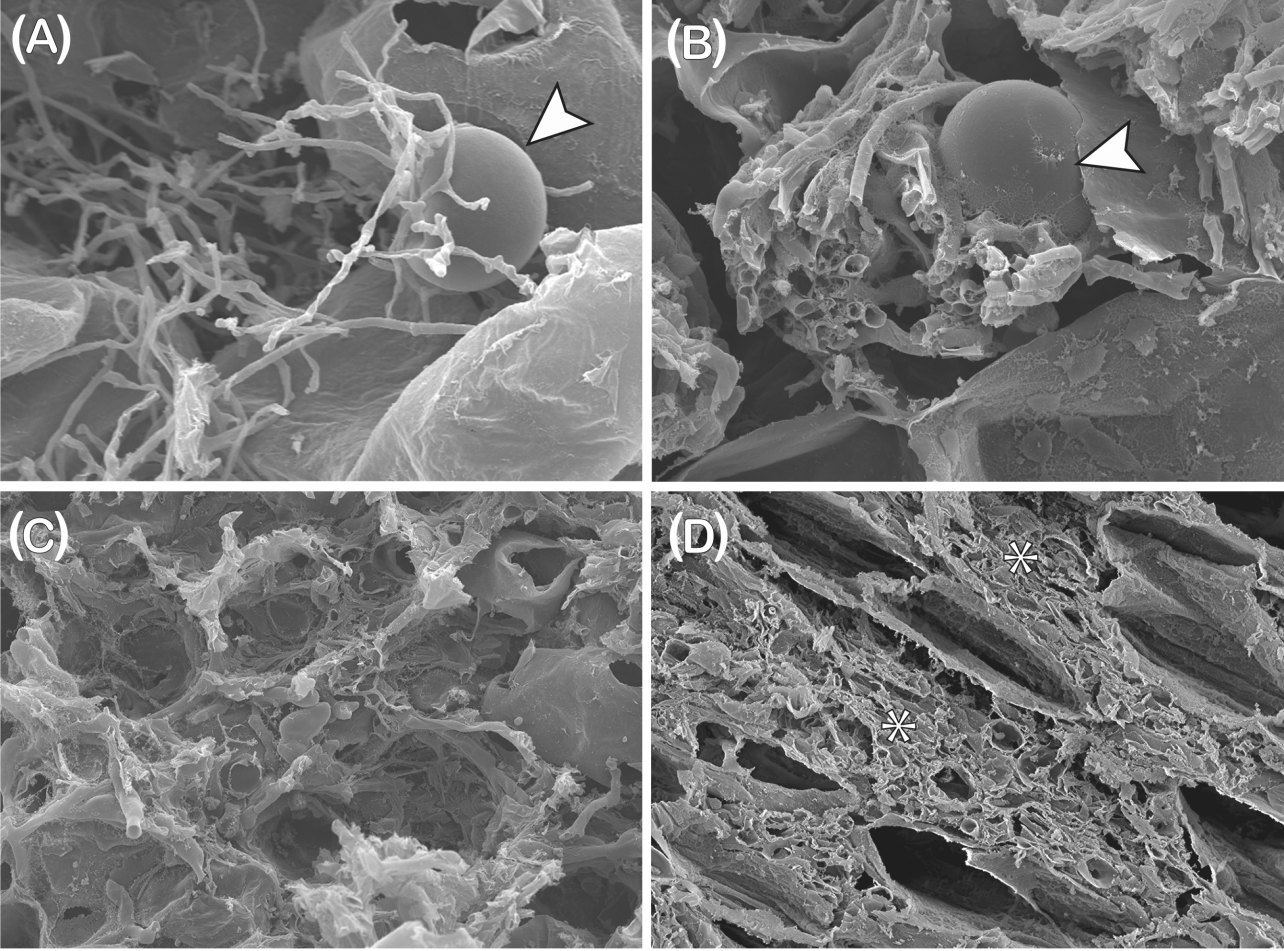
Scanning electron micrographs of structures produced by MFRE during colonisation of host plant tissues. (A) Intracellular small vesicle with fine, branching hyphae in the sporophytic protocorm of *Lycopodiella inundata*. (B) Intracellular tightly wound hyphal coil with larger vesicle in the outermost cortical layers of a gametophyte of *L. inundata*. (C,D) *L. inundata* sporophytic protocorm. During intercellular colonisation, the fine hyphae enlarge (C) until the intercellular spaces are filled with masses of collapsed pseudoparenchymatous hyphae (D*). Scale bars: (C) 50 μm; (B,D) 20 μm; (A) 10 μm

MFRE in angiosperms typically produce fine arbuscules together with fan‐like hyphal structures and hyphal ropes (Albornoz et al., 2020; Hoysted et al., 2019; Orchard, Standish, et al., 2017) and are distinguished morphologically from the ‘coarser’ AMF (Figure 1A,B) by their characteristic finer hyphae (<2 μm in diameter vs. >3 μm in AMF) with small intercalary and terminal vesicles or swellings (Orchard, Standish, et al., 2017; Figure 1C). Fine arbuscules (Figure 1D) alongside coarser ones have also been observed in liverworts co‐colonised by MFRE and AMF (Field et al., 2016, 2019) but, in this group and in other early‐divergent spore‐producing lineages, the morphology of colonisation by MFRE appears highly plastic (Field & Pressel, 2018; Pressel et al., 2021). Both intra‐ and intercellular phases of colonisation are present in the earliest divergent Haplomitriopsida liverworts and in the gametophyte and early sporophytic stage (protocorm) of several lycophyte species (Schmid & Oberwinkler, 1993; Duckett & Ligrone, 1992; Hoysted et al., 2019; Hoysted et al., 2020). Intracellular colonisation results in a variety of structures, including tightly wound hyphal coils with terminal swelling or “lumps” (Haplomitriopsida and outermost cortical layers of lycophyte gametophyte) (Figures 1E and 2B) and branched fine hyphae with intercalary and terminal vesicles (lycophyte gametophyte and protocorm) (Figure 2A) but, consistently, no arbuscule‐like structures (Carafa et al., 2003; Duckett et al., 2006; Hoysted et al., 2019). During intercellular colonisation, the fine hyphae enlarge (Figure 2C), eventually forming masses of swollen pseudoparenchymatous structures lacking vesicles and which soon collapse and degenerate (Figure 2D). This short life‐span mirrors that of the Haplomitriopsida intracellular fungal ‘lumps’. It has been suggested that the collapse and lysis of these structures (Figure 1F) may provide a source of nutrients, including N, passed from MFRE to their host liverworts, comparable to the previously discussed mechanism employed by OrMs (Duckett et al., 2006; Hoysted et al., 2020).

MFRE colonisation in lycophytes also varies depending on the plant's life stage. In the adult sporophytes of *Lycopodiella inundata*, colonisation is strictly intracellular and consists solely of fine branching hyphae with intercalary and terminal swellings (Figure 1C); as in earlier developmental stages, arbuscule‐like structures have never been observed in MFRE‐colonised roots of *L. inundata* (Hoysted et al., 2019; Hoysted et al., 2020). Recent demonstrations of nutrients‐for‐carbon exchange in Haplomitriopsida liverworts (Field et al., 2015) and adult sporophytes of *L. inundata* (Hoysted et al., 2019; Hoysted et al., 2020) by isotope tracer experiments indicate that arbuscules are not required for this exchange to occur and that other MFRE structures must therefore be involved in active metabolic interactions with the host cells. Several questions remain regarding the development, phenology, and functioning of MFRE structures in symbiosis with host plants (Box 1).

There is increasing evidence of a tightly controlled interplay between soil nitrogen and phosphorus dynamics and the characteristics of mycorrhizal colonisation and nutrient exchange with host plants. Low soil N and P, both individually and in combination, result in increased colonisation by the AMF *Rhizophagus irregularis* in the model legume *Medicago truncatula* (Chen et al., 2018). Starvation of these nutrients results in greater colonisation by mycorrhizal symbionts, likely due to the stress response induced by lack of these key nutrients (Bonneau et al., 2013). N and P interplay may also influence fungus to plant transfer of nutrients. The *M. truncatula* phosphate transporter mutant knockout line *Mtpt4* displays a premature arbuscule degeneration (PAD) phenotype; in plants supplied with low levels of N, PAD is suppressed (Javot et al., 2011). Suppression of PAD is dependent on the expression of the ammonium transporter AMT2;3 (Breuillin‐Sessoms et al., 2015), suggesting that the amount of N and P passed from the fungus to the plant is linked to soil N and P status. Despite the increasing evidence of the interaction between N and P nutrition in the dynamics of the symbiosis between mycorrhizal fungi and plants, the extent of this and the mechanisms underpinning it remain elusive. This is potentially further complicated by the apparent functional complementarity between MFRE and AMF, wherein MFRE may play a more significant role in host plant N nutrition with AMF playing a more significant role in plant P nutrition (Field et al., 2019). This could suggest that there is cross‐talk between the two symbionts or that the levels of each nutrient transferred by each symbiont are controlled by either the plant or nutrient source‐sink dynamics. Further research is urgently needed to explore this.

## 3 N SOURCE PREFERENCE

AMF are able to assimilate various forms of N, including urea, amino acids (AA), ammonium (NH_4_^+^), nitrate (NO_3_^−^), and various soluble proteins (Jin et al., 2012). There is some evidence that inorganic N is favoured over organic N when both are available (Whiteside et al., 2012), with NH_4_^+^ preferred to NO_3_^−^ (Johansen et al., 1996; Toussaint et al., 2004). This preference for NH_4_^+^ may decrease the energetically costly reduction of NO_3_^−^ by the fungus prior to assimilation (Marzluf, 1997). However, in some cases, it appears that the opposite preference is true (Ngwene et al., 2013; Thirkell et al., 2019), suggesting a degree of plasticity in N source preference in AMF.

Experiments have shown that both AMF and MFRE transfer N to their host plants (Fellbaum et al., 2012; Hawkins et al., 2000; Hoysted et al., 2019). Unlike the obligately biotrophic AMF, it is possible to culture MFRE axenically in the absence of a host plant, suggesting that these fungi have at least some facultative saprotrophic capabilities (Field et al., 2015). MFRE appear able to assimilate and transfer N from both organic (Field et al., 2019) and inorganic sources to their host plants (Hoysted et al., 2019), although the degree to which MFRE rely on organic N acquisition to maintain symbioses remains to be investigated (Box 1). Other mycorrhiza‐forming fungi also have saprotrophic capabilities; EcM fungi (Basidiomycota and Ascomycota) acquire both organic and inorganic forms of N, although they are likely to access organic sources more frequently in nature given how abundant (over 95% in some woodlands) organic material is in the woodland ecosystems where EcM are most common (Chalot & Brun, 1998; Nicolás et al., 2019). ErM are also capable of utilising nitrogen from both organic and inorganic sources; experiments using labelled tannins (polyphenol–protein complexes) showed N transfer to *Rhododendron maximum* (Makarov, 2019), while colonisation by the ErM *Rhizoscyphus ericae* increased the capacity of cranberry to absorb nitrate (Wei et al., 2016). The N source preference of *R. ericae* has been shown to depend on both the strain of the fungus and the availability of carbon (Grelet et al., 2005). Orchid mycorrhizas such as *Tulasnella calospora* have also been shown to assimilate both inorganic (ammonium nitrate) and organic (glycine) sources of N and transfer N to their hosts (Fochi et al., 2017). Based upon the nitrogen source preferences of other mycorrhizal fungi, it seems reasonable to assume that the preferred inorganic nitrogen source for MFRE would be ammonium, eliminating the energetic cost of reducing nitrate, although, like ErM, this may be dependent upon the availability of a carbon source to utilise for energy among other factors such as soil pH. While the direct mechanisms of N uptake in MFRE remain unknown, it is possible that MFRE may break down or assimilate organic N as well as assimilate inorganic N, thus resulting in the transport of substantially more N to host plants, per unit biomass, than AMF (Hoysted et al., 2019).

## 4 MECHANISMS OF MYCORRHIZAL‐MEDIATED N ASSIMILATION

**FIGURE 3:**
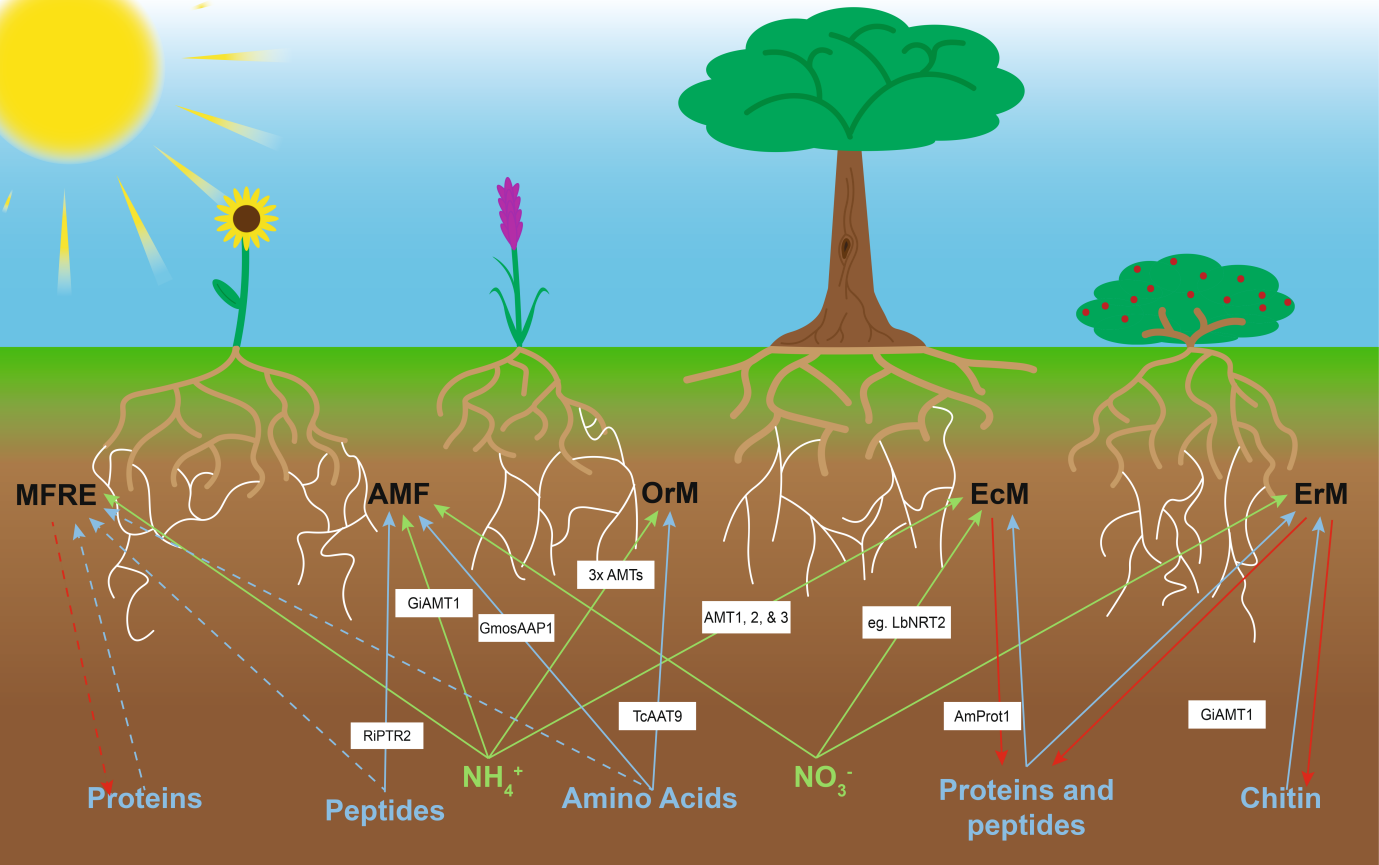
Forms of nitrogen present in soil and capabilities of mycorrhiza‐forming fungi to access and assimilate them with known key transporters and enzymes involved shown. Solid arrows represent known assimilation, whereas dashed arrows represent possible assimilation pathways. Red arrows indicate secretion of enzymes by fungi to degrade organic nutrient sources, green and blue arrows indicate inorganic and organic nitrogen sources, respectively (Belmondo et al., 2014; Cappellazzo et al., 2008; Fochi et al., 2017; López‐Pedrosa et al., 2006; Nehls et al., 2001; Stuart & Plett, 2020)

Ammonium (NH_4_^+^) uptake by AMF is achieved via transport proteins such as the high‐affinity ammonium transporter GintAMT1 expressed in the extraradical mycelium of *Glomus intraradices* in the presence of low concentrations of NH_4_^+^ (López‐Pedrosa et al., 2006; Calabrese et al., 2016; Figure 3). Similar NH_4_^+^ importers have been either functionally characterised or identified in a variety of mycorrhizal fungi, such as in the EcM species *Hebeloma cylindrosporum* and *Amanita muscaria* (Stuart & Plett, 2020) as well as the OrM species *Tulasnella calospora* (Fochi et al., 2017; Figure 3). MFRE are also capable of using NH_4_^+^ as a nitrogen source (Hoysted et al., 2019; Field et al., 2015; Figure 3) and although there are no genomic or transcriptomic data yet available to confirm this, it seems likely that, due to the commonality of NH_4_^+^ uptake mechanisms across the other groups of mycorrhizal fungi, MFRE also possess AMTs or similar NH_4_^+^ transporters. Nitrate (NO_3_^−^) uptake by AMF has been shown to occur via a H^+^ mediated symporter, GiNT, that is expressed in the extraradical mycelium (Bago et al., 1996; Tian et al., 2010). Similar nitrate transporters have also been found in the genomes of the EcM *H. cylindrosporum* and *Laccaria bicolor* (Stuart & Plett, 2020), suggesting that MFRE may also possess similar NO_3_^−^ transporters. However, an isolate of the OrM *Tulasnella calospora* lacks a nitrate uptake system (Fochi et al., 2017), perhaps indicative of a preference for organic N. Currently, N assimilation for MFRE has only been shown for NH_4_^+^ sources with the capacity for NO_3_^−^ assimilation and transfer representing a significant unknown (Box 1).

Although the capacity of AMF to assimilate organic forms of N remains ambiguous (Smith & Read, 2008), several potential mechanisms for organic N assimilation have been identified. The amino acid permease GmosAAP1, expressed by *Glomus mosseae*, is capable of actively importing proline via a H^+^ and pH‐mediated mechanism (Cappellazzo et al., 2008). It is expressed in both the intraradical and extraradical mycelium and transports multiple amino acids, including arginine, asparagine, and glutamine (Jin et al., 2005). In addition, the dipeptide transporter RiPTR2 has been shown to be expressed by *R. irregularis* in both the intra‐ and extraradical mycelium (Belmondo et al., 2014); however, the significance of this contribution to overall host plant N nutrition remains to be elucidated. A key deficiency in the ability of AMF to utilise complex organic nitrogen forms is that they do not appear capable of polymeric N degradation, relying instead on the breakdown of large organic compounds by other microbes in the rhizosphere (Chowdhury et al., 2022; Talbot & Treseder, 2010). It is possible that greater access to soil organic N pools derived from the saprotrophic capabilities of MFRE may result in the observed transport of substantially more N to host plants than AMF. Other mycorrhizal fungal groups with saprotrophic capabilities have complex mechanisms for accessing organic N; EcMs utilise a wide range of amino acids as both sources of N and C, and many of these fungi also often possess the enzymes necessary for the degradation of chitin (Nygren et al., 2007; Figure 3). EcM fungi secrete a diverse range of peptidases and have a number of amino acids, oligopeptides and dipeptide transporters that take up the resulting products, suggesting that protein is an important N source for these fungi (Nygren et al., 2007; Stuart & Plett, 2020). EcMs produce a range of different proteases, including aspartic proteases, serine proteases, metalloproteases, and cysteine proteases, such as the aspartic protease AmProt1 found in the EcM *Amanita muscaria* (Nehls et al., 2001). ErMs also produce enzymes capable of decomposing other complex organic molecules allowing, for example, mobilisation of N from chitin and polyphenol–protein complexes (Makarov, 2019). An amino acid transporter/permease (TcAAT9) has been found in the OrM *T. calospora* that is upregulated when provided with glutamine as the N source as opposed to ammonium, suggesting that OrMs are also capable of utilising amino acids as a N source (Fochi et al., 2017). Given their saprotrophic capabilities, it is plausible that MFRE may also possess a suite of degradative enzymes such as proteases that, in a manner similar to EcMs and ErMs, are secreted into the soil by transporters, allowing MFRE to assimilate the degradation products. Investigating into potential mechanisms of organic matter degradation using established methods (Shah et al., 2013) is now needed (Box 1). Once a genome for MFRE is available, comparisons between the suites of enzymes possessed by saprotrophic mycorrhiza‐forming fungi and MFRE will allow to predict the degradative capabilities of MFRE. This work can then inform further targeted study of putative degradation mechanisms using established techniques (Shah et al., 2013).

## 5 FUNGUS‐TO‐PLANT TRANSFER OF N

**FIGURE 4:**
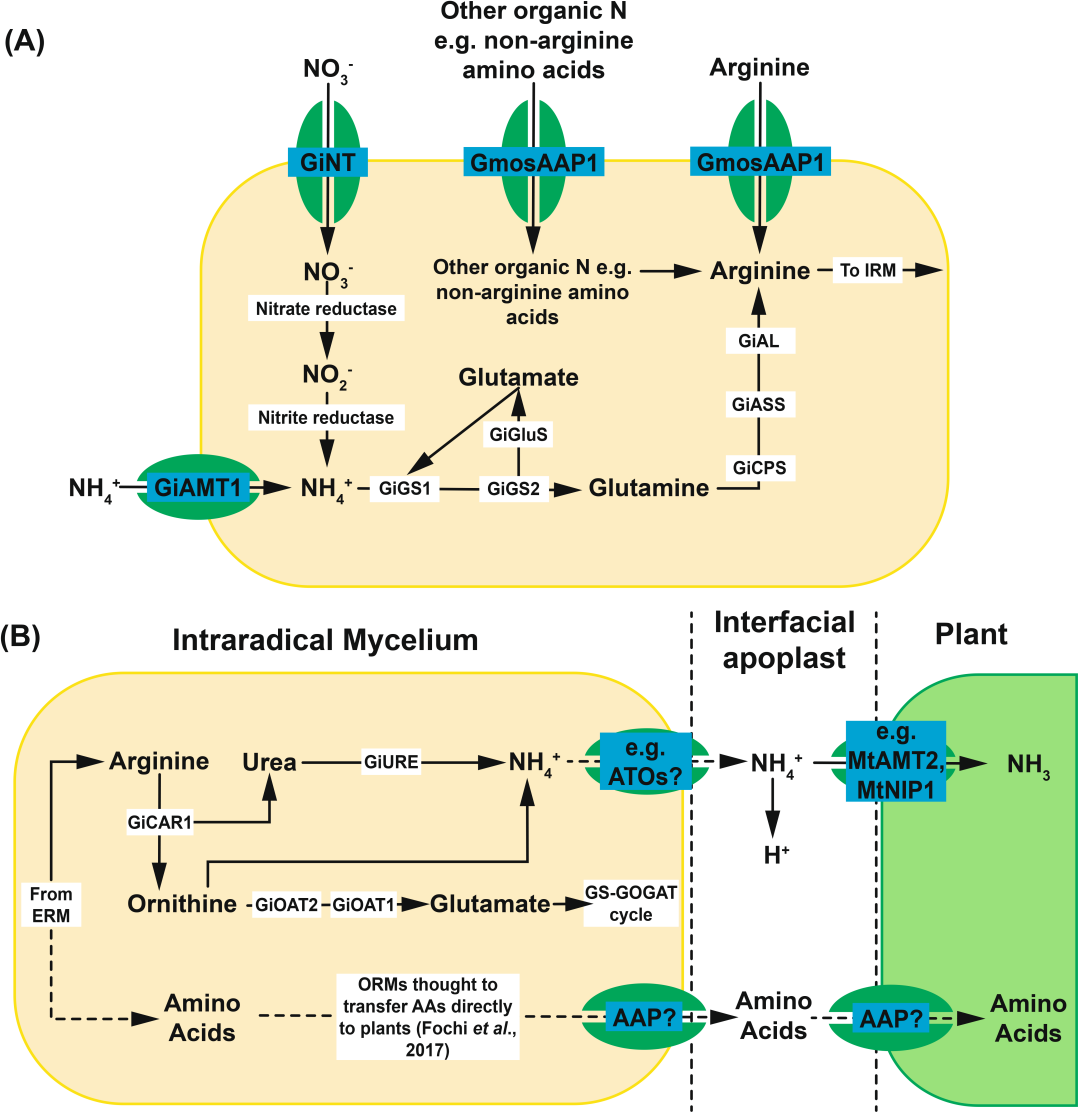
(A) Pathways by which nitrogen is assimilated and transformed within extraradical mycelium (ERM) of arbuscular mycorrhizal fungai (AMF) before export to host plants (Govindarajulu et al., 2005; Tian et al., 2010). (B) Pathways by which assimilated nitrogen is transferred from intraradical mycelium (IRM) to plant root cells. Solid arrows indicate known pathways. Dashed arrows indicate potential nitrogen assimilation pathway in orchid mycorrhizas (Chen et al., 2018; Cruz et al., 2007; Fochi et al., 2017; Govindarajulu et al., 2005; Jin et al., 2012)

After N is assimilated into AMF hyphae, it is converted to arginine for transport to the arbuscule; however, how N is processed into this form depends upon the original form of nitrogen imported (Jin et al., 2012). Nitrate is reduced to nitrite by nitrate reductase before being further reduced to ammonium by nitrite reductase (Jin et al., 2012; Figure 4a), which is then incorporated into arginine via the glutamine synthetase‐glutamate synthase (GS‐GOGAT) cycle (Govindarajulu et al., 2005). In *Rhizophagus irregularis*, GiGS1 and GiGS2 incorporate inorganic nitrogen into glutamate to produce glutamine, which is then converted into arginine (Tian et al., 2010; Figure 4a). Organic sources of N also require conversion into arginine for transport to the intraradical mycelium unless direct assimilation of arginine has occurred (Figure 4A). However, not all common amino acids are utilised by AMF. Cyclic amino acids and amino acids with high bond strengths resist hydrolysis and are consequently not efficient for use by AMF, regardless of size, and are thus assimilated less often (Talbot & Treseder, 2010).

Less is known about the processing of nitrogen once inside the extraradical mycelium of the other mycorrhizal types; in EcM neither the form of N transported to the intraradical mycelium nor the mechanisms behind this are fully known (Stuart & Plett, 2020). Wei et al. (2016) suggest that in the ErM *Oidiodendron maius*, nitrate is converted to arginine in a similar manner to AMF. Based on transcriptional evidence, it seems unlikely that the OrM *T. calospora* uses the arginine‐based transport pathway from extraradical mycelium to the intraradical mycelium since urease, which is required for the breakdown of arginine in the intraradical mycelium of AMF, is strongly downregulated in *T. calospora* (Fochi et al., 2017). Lack of a genome or transcriptomic data makes it difficult to speculate if arginine is the molecule used by MFRE to transport N from extraradical to intraradical mycelium. Because this appears to be a common mechanism shared by AMF and ErMs and given the phylogenetic proximity of MFRE to AMF, it is plausible that MFRE rely on a similar mechanism of N transport to that used by AMF.

Following conversion into arginine by AMF, N is transported from the extraradical mycelium into the intraradical mycelium to be exchanged with the plant (Fellbaum et al., 2012) (Figure 4B). Labelled substrate studies using ^13^C, ^14^C, and ^15^N have shown that only N is transferred between the fungus and the plant and that the C skeletons of amino acids synthesised by the fungus remain in the fungus (Govindarajulu et al., 2005); therefore, the arginine used to transport N to the intraradical mycelium must be broken down to release ammonium. Given that transcript levels of genes with high similarity to ornithine aminotransferase, urease accessory protein, and ammonium transporters are upregulated in the intraradical mycelium of *Rhizophagus irregularis* (Chen et al., 2018; Govindarajulu et al., 2005; Jin et al., 2012), arginine break down is likely to occur via the urea cycle, with amino acids recycled via the GS‐GOGAT cycle once ammonium has been liberated (Chen et al., 2018; Jin et al., 2012). Urease and arginase activity is increased in the mycorrhizal root compartment when compared with the extraradical mycelium, suggesting that the catabolic arm of the urea cycle is more active in the intraradical than the extraradical mycelium (Cruz et al., 2007; Jin et al., 2012).

A similar mechanism for arginine breakdown and ammonium release has been proposed for EcM fungi (Nehls & Plassard, 2018). Upregulation of plant ammonium importer expression in EcM mycorrhizal root tips indicates that ammonium is the principal form of N transferred between the two organisms (Stuart & Plett, 2020). Ammonium is also thought to be the principal form of N transferred by the ErM fungi to the plant, as shown in *Rhododendron fortunei* plants in symbiosis with *Oidiodendron maius* (Wei et al., 2016). When nitrate was provided as the N source, the expression of the plant ammonium transporter (*RfAMT*) increased threefold in colonised roots suggesting that, in a similar manner to AMF, nitrate is absorbed, reduced and converted into arginine before it is broken down in the intraradical mycelium to release ammonium for transfer to the plant (Wei et al., 2016). However, this does not appear to be the case for OrM. In colonised protocorms of *Serapias vomeracea* in symbiosis with *T. calospora*, both plant and fungal amino acid transporters were upregulated, whereas plant ammonium transporters were not strongly upregulated (Fochi et al., 2017). This suggests that an amino acid‐based, as opposed to ammonium‐based N transfer system, is used in this symbiosis.

In AMF, fungus‐to‐plant N transfer occurs at the periarbuscular interface between plant and fungal cell membranes (Figure 4B) although the mechanism by which fungal hyphae release N into the interfacial apoplast is currently unknown. Aquaporins may be involved as suggested by the identification of two fungal aquaporins expressed in the extraradical mycelium and arbuscules of maize roots inoculated with *R. irregularis* (Chen et al., 2018; Li et al., 2013). Recently, two *R. irregularis* genes with homology to *Saccharomyces cerevisiae AMMONIA TRANSPORT OUTWARD PROTEIN 3* (*ATO3*) have been identified; these could also be candidates for export of ammonia into the interfacial apoplast (Chen et al., 2018).

Uptake of nitrogen from the interfacial apoplast into the plant occurs via transport proteins such as the ammonium transporter GmAMT4.1 expressed in soybean root cortical cell membranes when in partnership with *Glomus intraradices* (Kobae et al., 2010). There are four other AM‐induced AMT genes identified in soybean, two in sorghum, two in tomato, and three in *Medicago truncatula* (Chen et al., 2018; Jin et al., 2012). In addition to ammonium transporters, nitrate transporters in the NRT2 family have been identified as AMF‐induced in tomato, *M. truncatula*, and *Lotus japonicus*, although their subcellular localisation and transport activities remain unknown and potential transfer of nitrate in the symbiosis is poorly understood (Hogekamp et al., 2011). Some organic forms of nitrogen may also potentially be transferred; AMF‐upregulated amino acid transporters have been observed in *L. japonicus* (Chen et al., 2018) although the labelled substrate studies mentioned above (Govindarajulu et al., 2005) would dispute this as a mechanism for transfer. In EcM, evidence of amino acid transport and the exchange of organic compounds in the apoplastic space also exists, with fungal amino acid exporters being upregulated in mycorrhizal root tips colonised by *Laccaria bicolor* or *Pisolithus microcarpus* (Stuart & Plett, 2020).

## 6 CONCLUSIONS AND FUTURE PERSPECTIVES

The reclassification of fine root endophytes as symbiotic Mucoromycotina fungi (Orchard, Hilton, et al., 2017) together with the significant differences in lifestyle and function when compared to AMF (Field et al., 2019; Lin et al., 2014), and developments in molecular identification (Bidartondo et al., 2011) throw into question much of what was assumed about the role of AMF in host plant N nutrition. Although structurally and phylogenetically closer to AMF (Hoysted et al., 2019; Spatafora et al., 2016), the apparent saprotrophic capabilities of MFRE (Lin et al., 2014) bring forth the hypothesis that some of the mechanisms these fungi use for nitrogen acquisition, transport and transfer to plants may be more similar to those of saprotrophic lineages of mycorrhiza‐forming fungi such as the EcM, ErM and OrM. The relatively wide host range of MFRE, similar to that of AMF (Orchard, Standish, et al., 2017), coupled with their increased access to organic N pools in the soil, suggests that their contribution to plant N nutrition across whole ecosystems could be vastly more significant than previously considered. Functional complementarity between MFRE and AMF (Field et al., 2019) with co‐colonisation by both symbionts commonly occurring across many plant species and ecosystem types (Hoysted et al., 2018; Orchard, Standish, et al., 2017) opens the possibility that in natural ecosystems with high levels of organic N MFRE may facilitate a significant proportion of plant N uptake.

The isolation and axenic culture of MFRE, and subsequent recolonisation of plants by the fungus (Field et al., 2015), provides an exciting potential experimental system by which the precise nutritional role of MFRE in symbiosis with plants both singly and in dual colonisations with AMF may be investigated. It also gives scope to providing the molecular and genetic tools to explore the mechanisms underlying nitrogen acquisition and processing, for example comparing the suites of enzymes MFRE may secrete to degrade organic matter with those of other saprotrophic mycorrhizal fungi, such as the EcM (Shah et al., 2013).

The interactions of MFRE with other soil microorganisms, such as rhizobacteria, add a further layer of complexity when exploring the role of MFRE in plant N nutrition. Likewise, AMF phosphate acquisition from organic sources is likely dependent on interactions with phosphate‐mineralizing bacteria associated with the hyphae (Jiang et al., 2021). Recent research has shown that different species of AMF colonising the same root system recruit distinct microbiomes around their extraradical mycelia with unknown impacts on soil nutrient cycling (Zhou et al., 2020). It is likely that MFRE also recruit their own distinct microbiomes, although the extent to which saprotrophic lineages require their microbiome to break down organic matter and transfer resulting nutrients on to the host plant requires further investigation. Again, isolation and axenic culture of MFRE, which do not have microbiome associates, will facilitate future avenues of research.

MFRE have a wide distribution across plant lineages and ecosystem types, from lycophytes in European heathlands (Kowal et al., 2020) to legumes in Australian pastures (Albornoz et al., 2021), with a potentially prominent role in host plant N nutrition (Field et al., 2019). Because of this, as well as experimental possibilities opened up by isolation and axenic culture experiments, future research into MFRE promises exciting new insights into plant N acquisition both at individual, community, and potentially even agricultural (Albornoz et al., 2022) levels. How this fits mechanistically and ecologically with other forms of mycorrhizas, involving both saprotrophic and obligately biotrophic fungi, is a key area for further study that can be facilitated by new molecular and bioinformatic tools.

## AUTHOR CONTRIBUTIONS

Katie J. Field, Silvia Pressel, and Tim J. Daniell conceived the concept and framing for the paper. Nathan Howard and Ryan S. Kaye wrote the first draft, Silvia Pressel constructed Figures 1 and 2, Nathan Howard drew Figures 3 and 4, all authors commented and developed subsequent versions of the manuscript and approved the final version.

## Data Availability

Data sharing is not applicable to this article as no new data were created or analysed in this study.
